# Origin, virological features, immune evasion and intervention of SARS-CoV-2 Omicron sublineages

**DOI:** 10.1038/s41392-022-01105-9

**Published:** 2022-07-19

**Authors:** Shuai Xia, Lijue Wang, Yun Zhu, Lu Lu, Shibo Jiang

**Affiliations:** 1 Key Laboratory of Medical Molecular Virology (MOE/NHC/CAMS), Shanghai Institute of Infectious Disease and Biosecurity, School of Basic Medical Sciences, Shanghai Frontiers Science Center of Pathogenic Microbes and Infection, Fudan University, Shanghai, China; 2 National Key Laboratory of Biomacromolecules, CAS Center for Excellence in Biomacromolecules, Institute of Biophysics, Chinese Academy of Sciences, Beijing, China

**Keywords:** Vaccines, Infectious diseases

## Abstract

Recently, a large number of severe acute respiratory syndrome coronavirus 2 (SARS-CoV-2) variants continuously emerged and posed a major threat to global public health. Among them, particularly, Omicron variant (B.1.1.529), first identified in November 2021, carried numerous mutations in its spike protein (S), and then quickly spread around the world. Currently, Omicron variant has expanded into more than one hundred sublineages, such as BA.1, BA.2, BA.2.12.1, BA.4 and BA.5, which have already become the globally dominant variants. Different from other variants of concern (VOCs) of SARS-CoV-2, the Omicron variant and its sublineages exhibit increased transmissibility and immune escape from neutralizing antibodies generated through previous infection or vaccination, and have caused numerous re-infections and breakthrough infections. In this prospective, we have focused on the origin, virological features, immune evasion and intervention of Omicron sublineages, which will benefit the development of next-generation vaccines and therapeutics, including pan-sarbecovirus and universal anti-CoV therapeutics, to combat currently circulating and future emerging Omicron sublineages as well as other SARS-CoV-2 variants.

## Introduction

On 24 November 2021, a novel SARS-CoV-2 variant (B.1.1.529) was first reported to the World Health Organization (WHO) and termed as the Omicron variant.^1^ Omicron was characterized with increased transmissibility accompanied by significant immune evasion. After only 2 days, WHO quickly classified it as a variant of concern (VOC), just like the Delta variant.^1^ Subsequently, Omicron further expanded into a series of sublineages,^2^ which were significantly different from those of previous SARS-CoV-2 variants. Recently, therefore, the origin, virological features, immune evasion and intervention of Omicron sublineages have attracted much attention.

## Origin of the Omicron variant

Although Omicron has caused another global epidemic,^1^ its origin remains unclear. Understanding its origin will provide crucial clues for effectively controlling it and avoiding other dangerous variants in the future.^3^ Some virologists suggested that Omicron might have diverged from other strains after long-term evolution through mutation or recombination in an isolated population with little surveillance.^3^ Based on GISAID data, early on, before its first discovered time point (9 November 2021) reported by WHO, Omicron samples had already been collected, but they were not sequenced and submitted until much later.^4^ Otherwise, the features of its decreased pathogenicity and S-gene target failure^5^ might have effectively improved estimates of its pathogenicity and transmissibility, even before its official confirmation. The timely sequencing and submission of positive samples is important for discovering the divergent variants and guiding the prevention and control of global SARS-CoV-2 epidemic. It is also possible that Omicron might have evolved in patients with chronic infection and immunodeficiency.^3,6^ For example, persistent infection of SARS-CoV-2 in an AIDS patient is related to the emergence of N501Y and D796Y mutations, which are also present in Omicron spike (S) protein.^3,6^ Therefore, global surveillance and sequencing of newly evolved variants in COVID-19 patients, particularly in immunocompromised individuals, is important for timely discovery and control of novel and dangerous variants.

The possibility of host-jumping in Omicron evolution has also been raised. Indeed, many animals are susceptible to SARS-CoV-2 infection. For example, SARS-CoV-2 can effectively infect white-tailed deer with a well-defined deer-to-deer transmission pathway.^7^ Mink-to-human transmission of SARS-CoV-2 in mink farms was reported.^8^ Similarly, the suspected cat-to-human transmission of SARS-CoV-2 in Thailand was also reported, where SARS-CoV-2 from the owner infected the cat, and was then transmitted to the veterinarian.^9^ Besides, many Omicron mutations, such as Q493R, Q498R and N501Y mutations in S protein, have been reported in adapted strains of mice, leading to the enhancement of viral adaptation and receptor engagement in mouse host, especially in aged mice,^10,11^ which could have served as an incubator of SARS-CoV-2 variants.^12^ Interestingly, both Omicron BA.1 and BA.2 S-trimers could bind with high affinity to mouse ACE2, while the ancestral SARS-CoV-2 S-trimer bound well to cat ACE2, rather than mouse ACE2, suggesting a possible human-cat-mouse-human evolution pathway for Omicron BA.1 and BA.2 sublineages.^12,13^ These phenomena suggested that Omicron predecessor might have undergone rapid evolution in the novel host environment and then back to humans.^14^ Therefore, the possibility of cross-species transmission of Omicron through host-jumping events deserves further investigation, and the potential animal reservoirs of SARS-CoV-2 and its variants should be rigorously monitored.

### The spread of Omicron sublineages

**Fig. 1:**
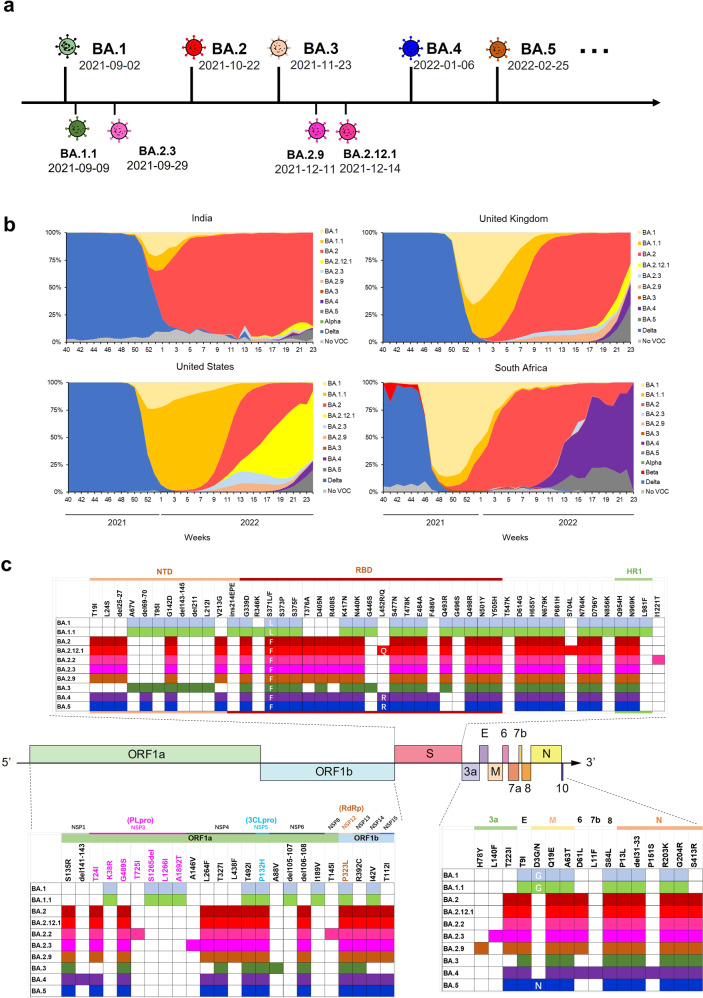
Emergence and spread of Omicron sublineages. **a** Emerging timeline for Omicron sublineages. Earliest date for each sublineage is from cov-lineages.org (continuously updated).^2^ **b** Prevalence of Omicron sublineages and other variants in India, the United Kingdom, the United States, and South Africa based on all sequences available on GISAID over the past 6 months. **c** Schematic representation of the genomic domains of SARS-CoV-2 with mutations in Omicron sublineages. PLpro (NSP3), papain-like protease; 3CLpro (NSP5), 3C-like protease; RdRp (NSP12), RNA-dependent RNA polymerase

From a global pandemic perspective, the Omicron variant has shown super transmissibility, rapidly replacing the Delta variant, which had been the dominant epidemic variant in many countries until the end of 2021.^15^ By 23 May 2022, over 3 million Omicron sequences had been submitted and then further divided into more than one hundred sublineages, such as BA.1 (original Omicron), BA.1.1, BA.2, BA.2.12.1, BA.2.3, BA.2.9, BA.3, BA.4, and BA.5^4^ (Fig. 1a). These sublineages also exhibit distinct capabilities of transmission and immune evasion. Notably, from February 2022, BA.2, which appears more transmissible, has become the most predominant strain in many countries, such as South Africa, the United Kingdom (UK) and India, quickly replacing BA.1 and BA.1.1^16,17^ (Fig. 1b). BA.2.12.1 also shows enhanced transmissibility and has become the dominant variant in the United States (Fig. 1b). However, BA.3 has shown limited transmissibility with few cases,^18^ suggesting that partial Omicron sublineages from random evolution only possess uncompetitive capacity in spreading. Therefore, systematic comparison of sequence differences between rare and dominant sublineages may reveal some critical mutations related to the currently circulating strains. BA.4 and BA.5 also emerged recently, already causing many infection cases in South Africa and spreading to many other countries^4^ (Fig. 1b). The future spread of BA.4, BA.5 and other sublineages should be closely monitored.

### Virological features of Omicron sublineages

Compared to ancestral SARS-CoV-2, Omicron sublineages contain numerous different amino acid sequences in their S proteins, nucleocapsid (N) proteins and other structural or non-structural proteins (Fig. 1c). Particularly, S protein, the most important functional protein for viral entry and infection, contains 31–37 mutations, many of which are shared among these sublineages: G142D in N-terminal domain (NTD) and G339D, S371L/F, S373P, S375F, K417N, N440K, S477N, T478K, E484A, Q498R, N501Y and Y505H in receptor-binding domain (RBD), as well as D614G, H655Y, N679K and P681H in S1 subunit and N764K, D796Y, Q954H and N969K in S2 subunit.

Some NTD mutations, including T19I, L24S, del25–27, G142D and del143-145, have caused significant evasion from NTD-targeted neutralizing antibodies (nAbs).^18^ Moreover, these shared mutations in RBD, combined with unique mutations, such as BA.4-specific L452R and F486V mutations or BA.2.12.1-specific L452Q mutations, play important roles in immune evasion, leading to numerous vaccine breakthrough infections and re-infections.^19^ Nevertheless, the hypermutated Omicron RBDs maintain their ACE2 receptor-engaging ability or even have increased binding affinity with ACE2.^20,21^ Compared to the S1 subunit, the S2 subunit, which contains heptad repeat 1 (HR1) and heptad repeat 2 (HR2) domains, has more conserved sequences. It also plays a key role in mediating viral fusion with and entry into the target cell through HR1-HR2 interaction to form six helix bundle (6-HB). BA.1-specific L981F mutation is located outside the HR1 fusion core.^22^ Similarly, Q954H and N969K in HR1, which are shared mutations in all Omicron sublineages, are located at the non-critical positions involved in HR1-HR2 interactions.^22^ Therefore, those HR1 mutations have little effect on 6-HB-mediated viral fusion and infection.

In addition, H655Y, N679K and P681H mutations are located at the S1/S2 border, which may affect the process of S1/S2 cleavage. Escalera et al. reported that the single H655Y mutation could significantly promote the cleavage and activation of S protein, thus enhancing viral fusogenicity.^23^ N679K and P681H mutations, with their similarity to the Delta-specific P681R mutation, are also speculated to be beneficial for viral fusogenicity.^24^ However, the whole Omicron-BA.1 S protein showed significantly decreased fusogenicity when compared to wild-type (WT) SARS-CoV-2.^25^ Some studies suggest that the weakened fusogenicity of Omicron-BA.1 is mainly related to reduced TMPRSS2-dependence in its entry process.^25^ Nevertheless, the in-depth mechanism of reduced fusogenicity mediated by BA.1-mutations remains to be further illuminated.

The fusogenicity of SARS-CoV-2 variants is positively correlated with their pathogenicity.^24,26^ For example, Delta variant with increased fusogenicity showed higher pathogenicity in patients. On the contrary, the BA.1 lineage with low fusogenicity exhibits milder pathogenicity in human lung tissue.^27^ Similarly, BA.2 also showed mild pathogenicity in mice and hamsters.^28^ However, a report has demonstrated that BA.2 S protein-mediated syncytia formation is more efficient than that of BA.1 S protein,^29^ suggesting that BA.2 may have gained enhanced pathogenicity during its evolution. A recent report also showed that the fusogenicity mediated by the S proteins of BA.2.12.1, BA.4 and BA.5 is significantly enhanced when compared to that of the original BA.1 lineage.^30^ However, compared with previously dominant variants, these Omicron sublineages showed reduced clinical pathogenicity, possibly through the effects of routine vaccination, which is expected to effectively attenuate disease severity, even though failing to prevent viral infection.^31^

In addition, some mutations are located at non-spike proteins, e.g., P13L, del31-33, R203K, G204R in N protein; T9I in E protein; Q19E and A63T in M protein; P132H in NSP5 (3C-like protease, 3 CLpro) and some mutations in ORFs. The potential effect of these non-spike mutations on viral transmissibility and pathogenicity is worthy of further study.^32^ For example, although the S protein of BA.3 could mediate vaccine immune evasion and cell entry comparable to that of BA.1 or BA.2,^33^ few BA.3 infection cases have been reported, possibly because of the non-spike mutations which result in the limited spread of BA.3. Notably, although BA.4 and BA.5 share the same mutant profile in their S proteins, they showed different spreading trends, according to the number of recently submitted sequences from South Africa (Fig. 1b). Therefore, further study of the roles of their non-spike mutations is warranted. For example, compared to BA.5, BA.4 contains several unique mutations, such as del141-143 in NSP1, L11F in ORF7b, and P151S in N protein. Thus, their potential effect on viral transmission needs further investigation.

### Immune evasion of Omicron sublineages

The most important feature of Omicron strains is their remarkable ability to evade immunity in convalescent COVID-19 patients and vaccinees.^19^ Numerous studies have reported that BA.1 and BA.2 could effectively reinfect convalescent COVID-19 patients.^16,34^ Moreover, BA.2.12.1, BA.4 and BA.5 bearing the lineage-specific L452Q/R mutation could cause significant humoral immunity escape.^21,35^ Previous study has shown that L452R is also related to cellular immunity evasion.^36^ Currently, several studies reported that BA.2.12.1, BA.4 and BA.5 could further escape from the immunity induced by BA.1 or BA.2 infections,^21,37^ resulting in numerous re-infections in many countries.

In terms of vaccine escape, current research shows that these approved vaccines, even with routine dosing, still failed to prevent the outbreak of Omicron variants.^38–44^ For example, original Omicron (BA.1) is markedly resistant to nAbs in sera from individuals who have received the routine dose of Ad26.COV2.S (vectored vaccine).^34^ Similarly, in BNT162b2 (mRNA vaccine) recipients, serum neutralizing potency against Omicron-BA.1 was decreased about 36–40-fold, even being completely ineffective in some vaccinees.^41,42,45^ Similar to BA.1, other Omicron sublineages, such as BA.2 and BA.3, also showed considerable vaccine immune evasion, mainly because of the numerous mutations in their S proteins.^46^ Besides, Omicron BA.1 and BA.2 are more prone to infect the human upper respiratory tract where systemic vaccination showed little efficacy of preventing viral infection,^47^ consistent with their enhanced transmissibility.

Fortunately, recent studies showed that the booster dose of homologous or heterologous vaccines, such as BNT162b2, mRNA-1273, or other approved vaccines, can significantly increase serum neutralizing titers against Omicron strains.^38,41–43^ For example, the booster dose of BNT162b2 could significantly improve neutralization efficiency against the original Omicron variant (BA.1).^48^ Similarly, the routine dose of mRNA-based vaccines could induce undetectable or very low titer of nAbs against Omicron variant, while sera from individuals who receive three or four doses of mRNA vaccine exhibited improved neutralization activity against Omicron sublineages.^49^ In addition, the symptoms caused by Omicron infection in vaccinees are relatively mild, possibly because of vaccine-induced cellular immunity.^50^ In general, vaccine-induced cellular immunity confers broader spectrum protection than the humoral immunity, particularly the protection against Omicron infections.^51,52^

### Intervention of Omicron sublineage infection with vaccines and therapeutics

Despite an already large prevalence, more Omicron sublineages with increased immune evasion are likely to emerge in the future, thus calling for the development of next-generation vaccines against the current circulating and future emerging Omicron sublineages. Recently, some Omicron-specific vaccines, such as the Omicron mRNA vaccine, have been reported to show higher efficiency against Omicron (BA.1) infection than WT mRNA vaccine.^42^ It was reported that Omicron infection could induce high titers of nAbs against other variants, such as Delta, in vaccinated, but not unvaccinated, individuals,^53^ suggesting that those new vaccines based on Omicron variant have the potential to serve as booster vaccines against homologous Omicron variant and other previous SARS-CoV-2 VOCs. However, it remains uncertain whether such vaccines could maintain their efficacy against emerging SARS-CoV-2 variants in the future. Most recently, it was reported that immune imprinting by previous SARS-CoV-2 exposure could significantly affect the immune boosting by Omicron infection.^54^ Therefore, pan-sarbecovirus vaccines based on multivalent or well-adjuvanted vaccines are being developed.^55–57^ For example, a vaccine based on Delta-Omicron chimeric RBD-dimer could elicit potent and broader neutralizing antibody responses in immunized mice against multiple SARS-CoV-2 variants.^58^ In addition, ferritin-based vaccines, such as S-trimer-ferritin nanoparticles, RBD-ferritin nanoparticles and multivalent ferritin vaccine, have been reported, which can induce broad and durable immunity against SARS-CoV-2 and its variants.^59,60^ A small-molecule non-nucleotide STING agonist-adjuvanted RBD-Fc vaccine was able to elicit extremely potent humoral and cellular immune responses against SARS-CoV-2 and its variants, as well as SARS-CoV and SARS-related CoV (SARSr-CoV) from bats.^55,56^ These pan-sarbecovirus vaccine strategies also provided new clues for the development of broad-spectrum vaccines against influenza viruses, which showed high mutation rates and severe immune evasion against routine vaccines.^61^ At present, most pan-sarbecovirus vaccines are still in the preclinical research stage and remain to be confirmed in clinical trials for their efficacy and safety in humans. In addition, in the development of next-generation vaccines, many important factors, including breadth, potency, long-acting effect and availability through nasal route, should be taken into consideration.

Antibody-based COVID-19 therapies have shown promising clinical safety and efficacy. Up to now, several nAbs have been approved by the Food and Drug Administration of the United States or European Medicines Agency for urgent treatment of mild to moderate COVID-19. Some of these nAbs, such as imdevimab and casirivimab, mainly target viral RBD and have shown potent neutralization activity against infection of WT SARS-CoV-2.^21,62–64^ However, Omicron variants show significant resistance to most of these approved nAbs, including imdevimab, casirivimab, bamlanivimab, etesevimab, regdanvimab and sotrovimab (Table 1). Among them, casirivimab plus imdevimab is no longer recommended for the treatment in Omicron-infected patients.^65^ Similarly, the Emergency Use Authorization (EUA) of sotrovimab was recently recalled owing to the ineffectiveness of its authorized dose against Omicron BA.2.^66^ Interestingly, cilgavimab, a potent neutralizing antibody against both WT SARS-CoV-2 and Delta VOC, retains potent neutralization activity against Omicron BA.2, BA.3, BA.4, and BA.5, but only mild potency against Omicron BA.1,^66^ indicating that some nAbs may regain their potency against other variants that will emerge in the future. Meanwhile, developing rapid diagnostic kits for the Omicron sublineages with which patients are infected is important for guiding the selection of antibody drugs.

**Table 1:** Neutralizing potency of approved nAbs for EUA against Omicron sublineages^21,62,64,67^

Approved nAbs	Authorized date stage	Neutralizing potency against SARS-CoV-2 or its variants		
		WT (614G)	Delta	Omicron sublineages
Imdevimab (REGN-10987)	November, 2020	Potent	Potent	Little efficacy against BA.1, BA.3 (IC_50_ > 5000 ng/ml) Moderate efficacy against against BA.2, BA.2.12.1, BA.4, BA.5 (IC_50_: 499–590 ng/ml)
Casirivimab (REGN-10933)	November, 2020	Potent	Potent	Little efficacy against BA.1, BA.2, BA.2.12.1, BA.3, BA.4, BA.5 (IC_50_ > 5000 ng/ml)
Bamlanivimab (LY-CoV555)	November, 2020	Potent	Little	Little efficacy against BA.1, BA.2, BA.2.12.1, BA.3, BA.4, BA.5 (IC_50_ > 5000 ng/ml)
Etesevimab (LY-CoV016)	February, 2021	Potent	Potent	Little efficacy against BA.1, BA.2, BA.2.12.1, BA.3, BA.4, BA.5 (IC_50_ > 5000 ng/ml)
Tixagevimab (COV2-2196)	December, 2021	Potent	Potent	Little efficacy against BA.1, BA.2.12.1, BA.3, BA.4, BA.5 (IC_50_ > 5000 ng/ml)
Regdanvimab (CT-P59)	September, 2021	Potent	Potent	Little efficacy against BA.1, BA.2 (IC_50_ > 5000 ng/ml)
Cilgavimab (COV2-2130)	December, 2021	Potent	Potent	Mild efficacy against BA.1 (IC_50_: 3007 ng/ml) Potent efficacy against BA2, BA.2.12.1, BA3, BA4, BA5 (IC_50_: 6.3–23 ng/ml)
Sotrovimab (S309)	May, 2021 to May, 2022	Potent	Moderate	Moderate efficacy against BA1, BA2, BA.2.12.1, BA3, BA4, BA5 (IC_50_: 361–989 ng/ml)
Bebtelovimab (LY-CoV1404)	February, 2022	Potent	Potent	Potent efficacy against BA1, BA2, BA.2.12.1, BA3, BA4, BA5 (IC_50_ < 5 ng/ml)
LY-CoV016 + LY-CoV555	February, 2021	Potent	Potent	Little efficacy against BA1, BA2, BA.2.12.1, BA3, BA4, BA5 (IC_50_ > 5000 ng/ml)
BRII-196 + BRII-198	December, 2021	Potent	Potent	Mild efficacy against BA1, BA3 (IC_50_: 1890–2190 ng/ml) Little efficacy against BA2, BA.2.12.1, BA4, BA5 (IC50 > 5000 ng/ml)

*Potent* IC_50_ < 100 ng/ml, *Moderate* IC_50_: 100–1000 ng/ml, *Mild* IC_50_: 1000–5000 ng/ml, *Little* IC_50_ > 5000 ng/ml; IC_50_: the half maximal inhibitory concentration

**Fig. 2:**
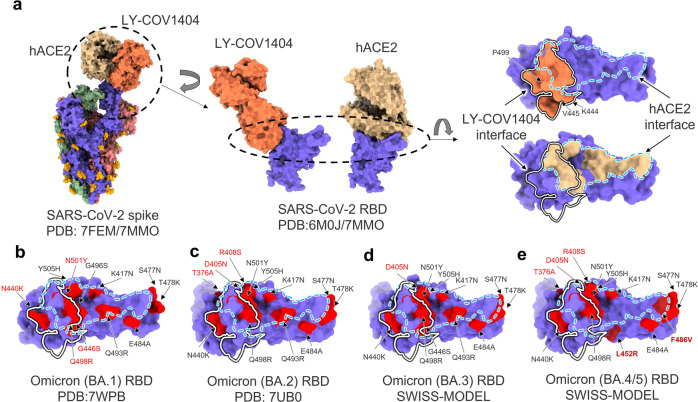
Structural comparations of ACE2-RBD interface and bebtelovimab (LY-CoV1404)-RBD interface in S protein of Omicron sublineages. **a** Superposition of complex structures of SARS-CoV-2 spike with human ACE2 (hACE2) receptor (PDB entry 7FEM) and spike RBD domain with LY-CoV1404 neutralizing antibody (PDB entry 7MMO) are shown on the left panel. RBD-LY-CoV1404 interface (PDB entry 7MMO) and RBD-hACE2 interface (PDB entry 6M0J) are enlarged in the middle panel and plotted on the RBD surface in the right panel. SARS-CoV-2 S protein is colored in medium slate blue, light coral and dark sea green for three protomers, respectively. Spike RBD domain alone is colored in medium slate blue. The hACE2 and its interface are colored in burlywood. LY-CoV1404 and its interface are colored in coral. Interface edges of LY-CoV1404 and hACE2 on RBD surface are indicated by white dotted line or blue dotted line, respectively. **b–e** Structural comparison of LY-CoV1404 binding interface, hACE2 binding interface and point mutations on spike RBD surface in Omicron sublineages, including BA.1 (PDB entry 7WPB) (**b**), BA.2 (PDB entry 7UB0) (**c**), BA.3 (predicted by SWISS-MODEL) and (**d**), BA.4/BA.5 (predicted by SWISS-MODEL) (**e**). RBD surface, interface edges of LY-CoV1404 and hACE2 are shown as (**a**). Point mutations of Omicron sublineages are colored in red, dark red or firebrick, and labeled accordingly

Notably, out of these clinical antibodies, bebtelovimab (LY-CoV1404) showed potent and broad neutralizing activity against infection by divergent Omicron sublineages, including BA.1 BA.2, BA.2.12.1, BA3 and BA.4/BA.5, as well as other SARS-CoV-2 VOCs.^21,67^ Based on structural analysis, bebtelovimab targets SARS-CoV-2 RBD with an epitope partially overlapping the ACE2-RBD interface. Therefore, the steric hindrance resulting from antibody binding effectively blocks RBD-ACE2 interaction^55,68^ (Fig. 2a). Three residues in RBD, K444, V445 and P499, are located in the center of bebtelovimab-RBD interface and make major contributions to the interactions (Fig. 2a). For the BA.1 sublineage, multiple mutations are located inside or around hACE2-RBD interface, which is consistent with its ability to escape vaccines. While no mutations are found in the bebtelovimab-RBD major interface, others like G446S, N440K, Q498R and N501Y are located at the edge of interface seem not to have a dominant effect on the neutralizing potency of bebtelovimab (Fig. 2b). Compared with BA.1, the BA.2 or BA.3 sublineage has additional T376A, D405N or R408S mutations, while BA.4 or BA.5 has more mutations of L452R and F486V, but all these mutations are distant from the bebtelovimab-RBD interface (Fig. 2c–e). These results suggest that bebtelovimab maintains potent neutralizing activity against all known Omicron sublineages. Mutational analysis has also proven that only K444Q, V445A or P499R/S mutation in S protein will lead to the significantly decreased activity of bebtelovimab.^63^ Fortunately, these mutations are very rarely presented in all current SARS-CoV-2 variants.

In addition, some small-molecule antiviral drugs also obtained EUA for COVID-19 treatment, and these still retain their efficacy against Omicron variants. For example, molnupiravir, an inhibitor of viral RNA-dependent RNA polymerase (RdRp), and nirmatrelvir, an inhibitor of viral 3CL protease, potently inhibited infection by the Omicron variant (specific sublineage not available) isolated from infected patients in vitro with IC_50_s similar to those of WT SARS-CoV-2.^69^ Moreover, Uraki et al. found that oral administration of both molnupiravir and nirmatrelvir considerably reduced virus titers in lung tissue in hamsters infected with BA.2.^28^ Nevertheless, their antiviral activity against other sublineages, such as BA.4 and BA.5, still needs further assessment. Besides, many preclinical antiviral agents, such as 1,5-anhydro-d-glucitol (1,5-AG),^70^ and the pan-CoV fusion inhibitors, such as EK1 and EK1C4,^71^ targeting the conserved site in S2 protein, also have good potential to broadly inhibit infection by SARS-CoV-2 variants and Omicron sublineages. For example, EK1 and EK1C4 can potently inhibit fusion mediated by S protein of WT SARS-CoV-2, Delta VOC, and Omicron BA.1. They could also significantly inhibit infection of pseudotyped and authentic Omicron variant.^71^ Considering the enhanced fusogenicity mediated by S protein of Omicron sublineages, pan-CoV fusion inhibitors like EK1 and EK1C4 may be superior over other inhibitors targeting the viral replication stages. Recently, Junqueira et al. and Sefik et al. reported that severe COVID-19 was closely related to SARS-CoV-2 infection of monocytes in the FcγR-dependent manner, which can cause systemic inflammation and severe COVID-19 pathogenesis,^72,73^ while Wang et al. showed that the fusion inhibitor EK1C4 could significantly prevent FcγR-mediated enhanced infection.^74^ Consequently, these pan-CoV fusion inhibitors may be particularly useful in protecting people from infection by SARS-CoV-2 Omicron sublineages and preventing the progression of inflammation-associated severe acute respiratory distress.

### Prospects

Recently, a large number of Omicron sublineages have continuously emerged, seriously threatening public health through vaccine breakthrough infections and reinfections. They have a significantly distinct immunological escape profile from that of ancestral SARS-CoV-2. Therefore, a bat coronavirus virologist proposed renaming these new Omicron sublineages as “SARS-CoV-3”.^75^ Evolutionarily, however, the sequence identity between ancestral SARS-CoV-2 and Omicron or its sublineages (BA.2, BA.2.12.1, BA.4 and BA.5) is more than 99.5%,^76^ which is much higher than that between SARS-CoV-2 and SARS-CoV-1 (79%)^77^ or MERS-CoV (55%).^78^ Therefore, this proposed renaming is contrary to the principles of nomenclature determined by the International Committee on Taxonomy of Viruses.^79,80^ Of course, we cannot exclude the possibility of emergence of the real SARS-CoV-3, a new species rather than a new variant, in the near future. It was reported that some SARS-CoV-2-infected patients were co-infected by MERS-CoV in Saudi Arabia.^81^ If this co-infection occurs in AIDS patients, emergence of SARS-CoV-3 is feasible through recombination by utilizing the identical Transcription Regulatory Sequences and/or cluster of sequence homologies at ORF1a and ORF1b in both SARS-CoV-2 and MERS-CoV.^82^

For the Omicron variant and its sublineages, many unsolved questions remain, including the reasons for their emergence, future evolutionary direction, and strategies for developing next-generation vaccines and therapeutics. Global surveillance and timely sequencing of novel SARS-CoV-2 variants are still of great importance currently. By targeting the conserved regions in spike protein, desigining multivalent antigens, and utilizing potent adjuvants, it is feasible to develop pan-sarbecovirus vaccines for prevention of the current or further Omicron sublineages as well as the outbreaks of the potentially emerged SARS-CoV-3 in the future.
